# Educational

**Published:** 1883-07

**Authors:** 


### Educational.

A suggestion was made in the June No. of the REGISTER that it would be pleasant, and might be profitable, for the faculties of the various Dental Colleges of the country, or their representatives, to meet for sociability and consultation at Niagara Falls, at the time of the meeting of the American Dental Association. Nearly all of the Colleges of the country have responded to that suggestion, approving most fully of the suggestion, and the general preference as to time for such a meeting seems to be on Tuesday evening, Aug. 7th, at the Cataract House.

It is here suggested that the attention of those interested in the matter be given somewhat to the subject in the meantime.

Perhaps Tuesday evening is as opportune a time as could be selected, It is much to be desired that every College in the country should be represented.

